# The impact of exercise self-efficacy, self-esteem and physical activity on body fat percentage changes in adolescents during fat loss interventions

**DOI:** 10.1038/s41598-026-37238-y

**Published:** 2026-01-23

**Authors:** Xiang Pan, Lupei Jiang, Yanfeng Zhang, Koya Suzuki, Yibo Gao, Jin He, Xiaoxiao Chen, Aoyu Zhang

**Affiliations:** 1 China Institute of Sport Science, Beijing, China; 2 Graduate School of Health and Sports Science, Juntendo University, Inzai, Japan; 3 Juntendo Administration for Sports, Health and Medical Science, Juntendo University, Tokyo, Japan; 4 Institute of Health and Sports Science & Medicine, Juntendo University, Inzai, Japan; 5 College of Physical Education and Sports Rehabilitation, Jinzhou Medical University, Jinzhou, China

**Keywords:** Exercise Self-Efficacy, Self-Esteem, Body fat percentage, Physical activity, Adolescents, Weight management, Lifestyle modification, Human behaviour

## Abstract

The prevalence of obesity among adolescents poses significant health threats. This study examines the predictive influence of self-efficacy (ESE), self-esteem (SE), and moderate-to-vigorous physical activity (MVPA) on the effectiveness of interventions aimed at reducing body fat percentage (BFP) among adolescents. A total of 100 adolescents were randomly assigned to the intervention group (IG) or the control group (CG). Baseline assessments measuring body composition, psychological indicators, and physical activity levels were conducted prior to the intervention. Intervention effects and group differences were evaluated using t-tests. Hierarchical regression models were constructed, using post-intervention BFP as the dependent variable (controlling for baseline). Following a 12-week structured intervention that included aerobic exercise, resistance training, and caloric restriction, The IG showed a reduction in BFP of 2.75% ± 1.90%, an increase in ESE of 12.14 ± 6.13 points, and an increase in SE of 8.58 ± 4.40 points. Hierarchical regression analysis in the IG revealed that increases in SE and MVPA were both significantly associated with negative changes in BFP (SE: *β* = -0.159, *P* < 0.001; MVPA: *β* = -0.079, *P* = 0.046). In the CG, no significant associations were found between changes in psychological factors and BFP after controlling for baseline levels. These findings underscore the pivotal role of psychological determinants in the effectiveness of weight loss interventions and suggest the need for an integrated approach that considers both physical activity and mental health to effectively manage obesity among adolescents.

**Supplementary Information:**

The online version contains supplementary material available at 10.1038/s41598-026-37238-y.

## Introduction

 The consequences of obesity in adolescents are multifaceted, making effective intervention during this critical developmental stage particularly important. The World Health Organization has defined obesity as a global epidemic metabolic disease that severely impacts human health, quality of life, and reduces life expectancy. In recent years, the incidence of overweight and obesity among children and adolescents in China has been increasingly alarming^1^. Conventional weight loss interventions typically include caloric restriction and physical exercise^2^; however, the influencing factors within these intervention contexts have not been thoroughly explored. Numerous factors that are highly correlated with changes in body fat percentage may play significant roles during the intervention process.

Exercise self-efficacy (ESE) refers to an individual’s confidence in their ability to perform specific tasks in physical activities, and this concept is a core element of social cognitive theory^3^. While broadly defined, for adolescents specifically, ESE represents the capacity to organize and execute physical activity even in the face of unique difficulties such as academic stress^4^. Research shows a protective association between high ESE and metabolic syndrome-related obesity^5^. However, unlike adults, adolescents are in a developmental stage marked by a conflict between autonomy and social reliance, where high ESE serves as a crucial resource to sustain healthy behaviors^4^. Beyond task-specific confidence, global self-evaluations such as self-esteem (SE) also significantly influence health behaviors. Significant correlations exist between physiological changes and variations in body esteem within interventions^6^. SE negatively correlates with total cholesterol and low-density lipoprotein levels in adolescents^7^.

By synthesizing Self-Determination Theory with Self-Efficacy Theory, this research postulates that ESE and SE function as internal psychological resources that translate intervention-driven motivation into sustained behavioral adherence. For instance, research on the motivations of low-income Latino communities participating in free community weight loss programs in border regions found that extrinsic motivations and monetary incentives are the primary driving forces for participants’ weight loss, while intrinsic motivation is relatively lacking. Strategies aimed at enhancing intrinsic motivation may help improve the effectiveness of weight loss interventions^8^. Specifically, the theoretical pathway suggests that physical activity improves health outcomes primarily by enhancing intrinsic factors like self-esteem^9^. In this study, we adopt Rosenberg’s widely cited definition of SE^10^, which defines SE as a positive or negative attitude toward oneself. Moreover, exercise habits are also critical factors influencing changes in body fat percentage. Studies indicate that physical activity levels are closely related to factors such as gender, age, BMI, education level, income, region, and urbanization. In normal-weight males, an increase of 4.5 MET·h/d in physical activity is associated with a decrease in body fat percentage; this negative correlation is even more pronounced in normal-weight obese populations^11^. Complementing these adult findings, longitudinal evidence from the COVID-19 pandemic highlights that for adolescents, regular physical activity protects against psychological distress over time by maintaining high levels of self-esteem^12^.

While existing research identifies ESE and SE as key predictors of weight loss success^13,14^, most evidence originates from adult populations or relies on cross-sectional designs^15^, limiting generalizability to adolescents and precluding causal inferences. Furthermore, while recent adolescent studies suggest that physical activity enhances mental health through improved SE^16^, these investigations often prioritize psychological outcomes over physiological body composition. This leaves a critical gap in understanding how the interplay between psychological factors and MVPA drives body fat changes during structured interventions. Consequently, the insufficient empirical focus on these interactions in adolescents compromises the development of targeted weight-loss strategies.

To address these research gaps comprehensively and thoroughly, drawing on the theoretical framework and preliminary findings described above, this study aims to investigate the effects of a 12-week comprehensive exercise intervention on changes in BFP (ΔBFP) among Chinese adolescents, and to analyze the roles of ESE, SE, and MVPA in influencing ΔBFP during the intervention. Thus, the following hypotheses were formulated:

H1: After the 12-week comprehensive exercise intervention, adolescents in IG will demonstrate significantly decreased BFP and significantly improved ESE and SE compared to those in CG.

H2: Within the IG, changes in ESE, SE, and MVPA (ΔESE, ΔSE, ΔMVPA) will be significantly associated with ΔBFP.

H3: Changes in ESE, SE, and MVPA (ΔESE, ΔSE, ΔMVPA) will significantly predict ΔBFP, and predictive effects of these factors will differ between the IG and CG.

## Methods

### Procedure

This study was a prospective randomized controlled trial in which 100 participants were recruited and randomly assigned to either an IG (*n* = 50, 25 males, 25 females) or a CG (*n* = 50, 24 males, 26 females). Prior to the intervention, all recruited subjects provided informed consent, consent for questionnaire participation, and agreement to privacy protection. Baseline measurements of morphological indicators, body composition, physical activity, ESE, and SE were conducted. The IG underwent a 12-week weight loss intervention comprising aerobic exercise, resistance training, and dietary caloric restriction. The exercise sessions were scheduled for 4:30 PM to 5:30 PM, three times per week. Specific interventions are listed in Supplementary File 1. The CG continued with regular school education and activities without any targeted interventions, thereby maintaining their usual lifestyle to ensure comparability between the IG and CG under identical conditions. The effects of the intervention were evaluated and analyzed for both groups, with a focus on the changes in BFP as the primary outcome measure.

### Subjects

#### Sample size calculation

Sample size calculations were performed using G*Power, with the primary outcome being the reduction of BFP. To ensure adequate statistical power, we established a power level of 0.80 (*β* = 0.20) and a significance level of *α* = 0.05. Based on a meta-analysis^17^, the correlation between pre- and post-intervention was assumed to be 0.80, with an effect size of 0.32, resulting in a calculated required sample size of 12 participants per group. However, accounting for potential attrition, we increased the sample size by 20%, leading to a required sample size of 14 per group. Considering gender as a variable for differential analysis, the minimum total sample size required was 56 participants. Additionally, taking into account the significance level for multiple regression analyses, the number of independent variables, the anticipated explanatory power of the model, and potential attrition, both the IG and CG required a minimum of 50 recruits each, yielding a total of 100 participants.

#### Recruitment and screening procedure

The study population was recruited from middle school students in Beijing. Recruitment of participants began on August 10, 2022, and ended on September 1, 2022.

Initial health screenings were conducted, with inclusion criteria as follows: (1) age between 13 and 15 years; (2) assessed as physically healthy through the Physical Activity Readiness Questionnaire (PAR-Q); (3) no history of professional athletic experience; (4) ability to comprehend the testing procedures, willingness to participate in the entire assessment process, and provision of informed consent. Exclusion criteria included: (1) severe organic lesions of the cardiovascular, neurological, pulmonary, renal, or musculoskeletal systems; (2) ongoing medication for chronic illnesses; (3) history of mental illness; (4) inability to complete follow-up or demonstrated poor compliance.

#### Group allocation and randomization

Participants were randomized using block randomization. Specifically, participants were first stratified by gender and age, and subsequently randomized within each stratum into either the IG or CG at a ratio of 1:1 using computer-generated randomization. To ensure there were no significant differences between groups in demographic characteristics and baseline measures (including age, gender, BMI, and initial BFP), we conducted randomization carefully.

### Assessment of indicators

Height and weight were measured using an electronic height and weight scale. Body composition was assessed using the bioelectrical impedance method (InBody 3.0, South Korea), from which BFP, fat mass, and fat-free mass were derived. To minimize measurement errors, all assessments were conducted in a fasted state at the same time of day, and participants were instructed to avoid strenuous physical activity prior to testing. All tests were conducted at the school’s Physical Fitness Centre.

The assessment of SE is based on the widely accepted 10-item scale^18^. The measurement of ESE was conducted using a validated ESE questionnaire^19^. This questionnaire is a reliable and effective instrument for assessing individuals’ confidence in their exercise capabilities and can be utilized to evaluate self-efficacy in exercise behavior^3,20^. Subjects were instructed by the researcher to complete the questionnaire. In the present study, Cronbach’s alpha coefficients for the SE and ESE questionnaires were 0.765 and 0.741, respectively, while the corresponding RMSEA values were 0.065 and 0.059, respectively.

MVPA refers to physical activities conducted at a moderate intensity or higher. The Actigraph accelerometer provides a more precise assessment of an individual’s activity level by detecting variations in acceleration^21^. Participants were instructed to wear the Actigraph GT3X + securely on their left wrist to ensure proper fit. They were required to wear the accelerometer throughout the day and record a minimum of four days of activity, including at least one weekend day, for the data to be included in the analysis. The calculation for MVPA time was as follows: [(average weekday MVPA * 5) + (average weekend MVPA * 2)]/7.

### Statistical analysis

Statistical analyses were performed using SPSS 26.0. Data are presented as mean ± standard deviation (Mean ± SD). Independent samples t-tests were used to compare baseline characteristics between groups. Analysis of Covariance (ANCOVA) was employed to evaluate intervention effects on primary outcomes, adjusting for baseline values to control for any pre-existing differences between groups. Paired samples t-tests were used to assess within-group changes. To rigorously assess the impact of changes in psychological and behavioral factors on body fat while controlling for regression-to-the-mean effects, we employed hierarchical linear regression analysis. The post-intervention BFP (T1) was set as the dependent variable. In the first block (Model 1), baseline BFP (T0), age, and gender were entered as control variables. In the second block (Model 2), the changes in SE, ESE, and MVPA were entered. Model fit was evaluated using F-values, *R*^2^, and △*R*^2^ statistics. Collinearity was assessed using Variance Inflation Factor (VIF), and residual plots were inspected to ensure assumptions of homoscedasticity and normality were met. Statistical significance was set at *P* < 0.05.

### Declarations

The study protocol was approved by the Ethics Committee of the China Institute of Sport Science (approval number: CISSLA20220801). All participants and their legal guardians provided written informed consent prior to participation. The informed consent document clearly described the research objectives, procedures, potential risks and benefits, participant rights (including voluntary participation and withdrawal at any point during the study), and confidentiality measures for personal information. All data were processed anonymously to ensure participants’ privacy. Furthermore, this study was registered with ClinicalTrials.gov (registration number: NCT06524908, Date: 2024-07-29) and was conducted in strict accordance with the ethical principles outlined in the Declaration of Helsinki.

## Results

### Descriptive statistics

**Fig. 1:**
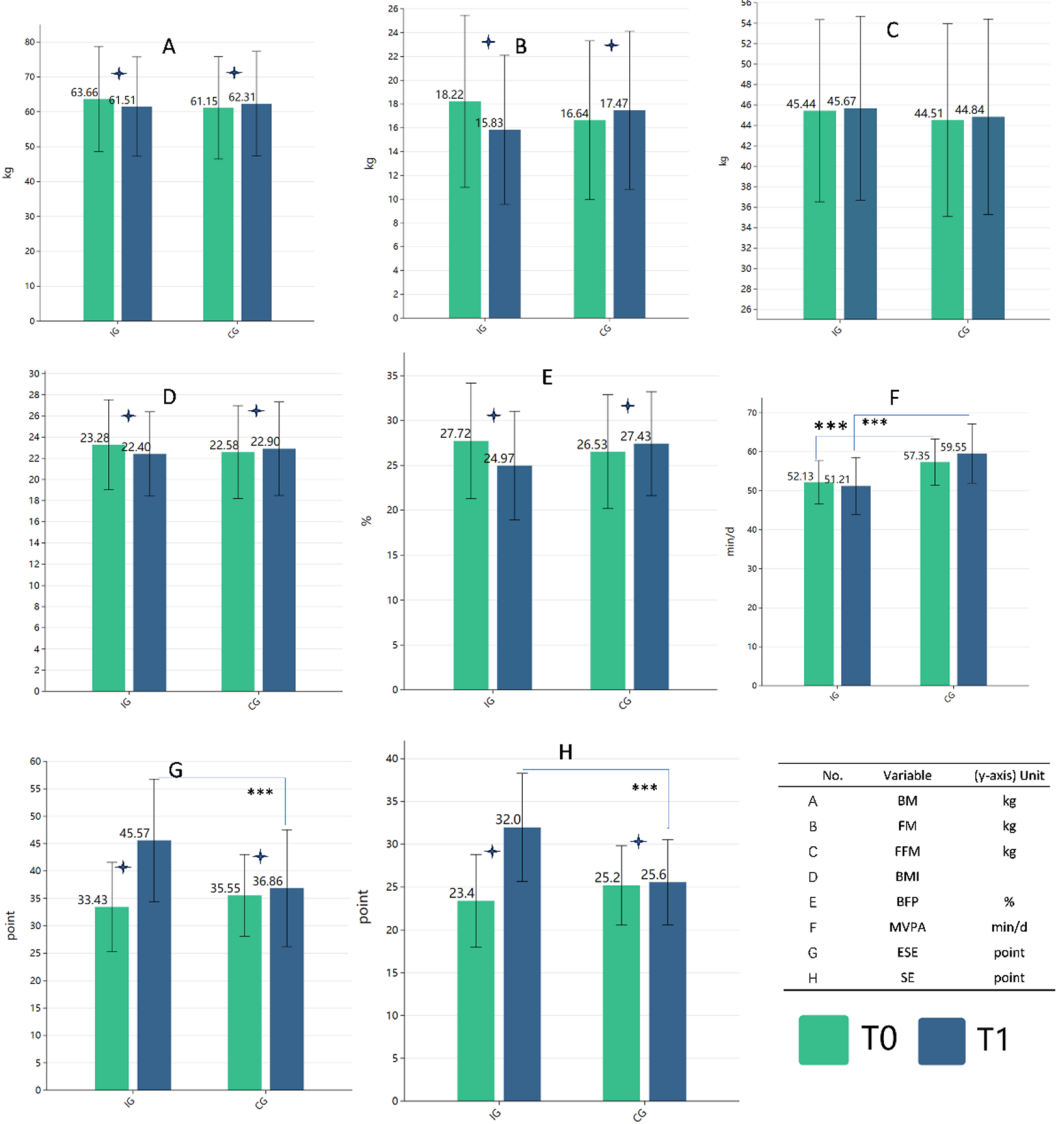
Changes in various variables over time for the intervention group (IG) and control group (CG) at baseline (T0) and after 12 weeks (T1). **Note**: Changes in various variables from baseline (T0) to after 12 weeks (T1) for the intervention group (IG) and control group (CG). Error bars represent standard deviations. Variables: (**A**) Body mass (BM, kg); (**B**) Fat mass (FM, kg); (**C**) Fat-free mass (FFM, kg); (**D**) Body mass index (BMI); (E) Body fat percentage (BFP, %); (F) Moderate-to-vigorous physical activity (MVPA, min/day); (G) Exercise Self-Efficacy (ESE, points); (H) Self-Esteem (SE, points). (+) indicates a significant within-group difference (paired sample t-test, *P* < 0.05) from baseline (T0) to 12 weeks (T1). (***) indicates a significant between-group difference (Independent samples t-test, *P* < 0.001) at the corresponding measurement time (T0 or T1). IG = Intervention group; CG = Control group; T0 = Baseline; T1 = Post 12-week intervention.

During the study, one female participant from the IG and one male participant from the CG withdrew for personal reasons. Consequently, the final sample size that completed the study and provided complete data was 98 participants, distributed as follows: the IG consisted of 49 participants (25 males and 24 females), all of whom attended every session and completed a total of 36 training sessions during the 12-week intervention, and the CG comprised 49 participants (23 males and 26 females). Descriptive statistics of the baseline data are presented in Table 1. Independent samples t-tests indicated that there were no significant differences between the IG and CG in terms of age, morphological characteristics, or psychological indicators, except for baseline MVPA which was significantly higher in the IG. Paired-samples t-tests demonstrated significant pre- and post-intervention differences within groups (Fig. 1). The IG showed a significant reduction in BFP of 2.75% ± 1.90%, an increase in ESE of 12.14 ± 6.13 points, and an increase in SE of 8.58 ± 4.40 points compared to the CG. We calculated the delta value (Δ = T1 − T0) to proceed with further analyses.

### Factors influencing BFP changes in the intervention group

To strictly control for the impact of baseline values on outcomes, we employed a hierarchical regression analysis with post-intervention BFP (T1) as the dependent variable, while controlling for baseline BFP (T0), age, and gender. The results are presented in Table 2. Model 1, which included demographic and baseline variables, explained 91.8% of the variance, with baseline BFP being the strongest predictor (*β* = 0.927, *P* < 0.001). In Model 2, the inclusion of change scores (△ESE, △SE, △MVPA) significantly improved the model’s explanatory power (△*R*^2^ = 0.044, *P* < 0.001). Specifically, increases in Self-Esteem (△SE) were significantly associated with lower post-intervention BFP (*β* = −0.159, *P* < 0.001), as were increases in MVPA (*β* = −0.079, *P* = 0.046). However, changes in Exercise Self-Efficacy (△ESE) did not show statistical significance in this model (*P* = 0.572). Collinearity diagnostics indicated no issues, with VIF values for all variables remaining below 1.7.

### Factors influencing BFP changes in the control group

Using the same analytic strategy for the Control Group (Table 3), Model 1 revealed that baseline BFP was the primary predictor of post-intervention BFP (*β* = 0.982, *P* < 0.001). However, unlike the Intervention Group, the addition of change scores in Model 2 did not significantly increase the explained variance (△*R*^2^ = 0.002, *P* = 0.467). None of the psychological or behavioral change variables (△SE, △ESE, △MVPA) showed a significant association with post-intervention BFP (*P* > 0.05). This suggests that without the structured intervention, natural fluctuations in these factors did not translate into significant changes in body composition.

## Discussion

Self-esteem increased in the intervention group. Low self-esteem has been linked to limited parental support and adverse life events during childhood/adolescence^22^ and is also associated with higher BMI in youth and later adulthood^23^. Longitudinal evidence indicates that adolescents with obesity are more likely to report low self-esteem in adulthood^24^. Participation in physical exercise may enhance self-esteem partly by promoting supportive social interaction, a key determinant of self-esteem^25–27^.

Baseline BFP was a robust predictor of the magnitude of BFP reduction induced by the intervention, with individuals starting at higher levels of adiposity exhibiting more pronounced responses. This finding aligns with previous human studies showing that intervention efficacy is closely linked to initial obesity status^28^.

In the intervention group, hierarchical regression (adjusting for baseline BFP, age, and gender) indicated that increases in self-esteem (ΔSE) were independently associated with lower post-intervention BFP. This pattern is consistent with prior work linking lower self-esteem to unfavorable body-composition profiles^29^ and suggests that improvements in self-esteem may support healthier behaviors during weight-loss programs. One possible mechanism is that higher self-esteem facilitates adherence and self-regulation and reduces maladaptive coping. Although exercise self-efficacy increased after the intervention, changes in ESE were not independently associated with post-intervention BFP in the multivariable model. ESE may primarily influence proximal behaviors and its effect on adiposity may be indirect, which could attenuate its independent association when these variables are modeled together.

Changes in MVPA were also independently associated with lower post-intervention BFP, which aligns with evidence that MVPA is inversely related to adiposity in adolescents^30–33^. Even relatively small increases in MVPA can contribute to a sustained negative energy balance and measurable reductions in body fat. In this study, the supervised protocol likely helped ensure that activity intensity and frequency were sufficient to influence body composition.

In the control group, changes in self-esteem, exercise self-efficacy, and MVPA were not significantly associated with post-intervention BFP after adjustment for baseline BFP, suggesting that natural fluctuations in these factors were insufficient to produce measurable body-composition changes without a structured program. Although ESE may support exercise motivation and adherence even in non-intervention contexts^3,34^, the present findings indicate that a structured program may be necessary to translate psychological changes into a sustained behavioral dose that is sufficient to influence body composition.

Overall, the findings suggest that a comprehensive exercise program combined with caloric restriction can reduce adolescent body fat percentage while improving ESE and SE. It is therefore recommended that schools and healthcare institutions implement comprehensive intervention programs, integrating not only physical activity and dietary strategies^35^ but also psychological support to achieve long-term intervention effects. Given the moderating roles of initial body fat levels and psychological factors on intervention outcomes, comprehensive assessments prior to intervention are advised. Accordingly, schools and healthcare institutions should design individualized and adaptive exercise programs and dynamically adjust intervention strategies based on personal characteristics, ensuring precise, targeted, and effective intervention outcomes.

Taken together, this study provides evidence that the exercise intervention reduced adolescent body fat percentage while concurrently enhancing ESE and SE. It is therefore recommended that schools and healthcare institutions implement comprehensive intervention programs that integrate physical activity and dietary strategies, while also incorporating appropriate psychological support to promote adherence and longer-term benefits. Given the moderating roles of baseline body fat and psychological factors in intervention outcomes, comprehensive assessments prior to intervention are advised. Accordingly, schools and healthcare institutions should design individualized and adaptive exercise programs and adjust intervention strategies based on participants’ characteristics to improve precision and effectiveness.

In this study, we included an inactive control group that continued usual school activities to evaluate the effectiveness of the intervention and to develop separate models for examining factors associated with post-intervention BFP under intervention and usual-lifestyle conditions. This approach helped to clarify how these factors may operate in different contexts. However, several limitations concerning sampling, measurement validity, and residual confounding should be acknowledged. Due to practical constraints, the study used an inactive control group, which makes it difficult to fully exclude potential expectancy or participation effects; future studies should consider adding an active control condition to better isolate intervention-specific effects. Participants were recruited from a single school, which may limit generalizability. Baseline MVPA was higher in the intervention group, which may have influenced group comparisons despite statistical adjustment. Although considerable effort was made to control confounding variables, dietary intake outside school was not directly quantified and pubertal status was not assessed, which may contribute to residual confounding. Regarding measurement validity, InBody 3.0 is widely used due to its convenience, but it is less precise than dual-energy X-ray absorptiometry and can be influenced by hydration status. Psychological factors were assessed via self-report questionnaires, which are susceptible to social desirability bias. Participants lived and studied in the same environment, which may have introduced a Hawthorne effect and potentially influenced participants’ behaviors or the study outcomes. Future research should employ more rigorous designs and measurements to further validate these findings and clarify the underlying mechanisms.

## Conclusions

This study confirms the critical role of ESE, SE, and MVPA in the effectiveness of fat loss interventions among adolescents. Considering both physical activity and mental health can enhance intervention outcomes more effectively. Furthermore, promoting intrinsic motivation and self-efficacy may facilitate sustained participation in healthy behaviors. Long-term research should explore the interplay of various factors influencing adolescent health behaviors to develop practical and effective comprehensive obesity management strategies.

**Table 1:** Baseline demographic, morphological, psychological, and behavioral characteristics of the participants (Mean ± SD or *n* (%)).

Indicators		Intervention group (*n* = 49)	Control group (*n* = 49)	Total	*P*
Age (years)		13.84(0.77)	13.9(0.74)	13.87(0.76)	0.690
Gender	Male	25(51.0)	23(46.9)	48(49.0)	
	Female	24(49.0)	26(53.1)	50(51.0)	
Morphology	Height (cm)	164.7(8.2)	164.1(8.3)	164.4(8.2)	0.732
	Weight (kg)	63.7(15.1)	61.2(14.7)	62.4(14.9)	0.405
	Fat free mass (kg)	45.4(8.9)	44.5(9.4)	45.0(9.1)	0.619
	Body fat mass (kg)	18.2(7.2)	16.6(6.7)	17.4(7.0)	0.261
	BFP (%)	27.7(6.4)	26.5(6.3)	27.1(6.4)	0.358
	BMI	23.3(4.20)	22.58(4.38)	22.93(4.31)	0.420
Psychological	ESE	33.4(8.2)	35.6(7.5)	34.5(7.8)	0.182
	SE	23.4(5.4)	25.2(4.6)	24.3(5.1)	0.078
Behavioral	MVPA (min/d)	57.35(5.91)	52.13(5.57)	54.74(6.28)	0.000

Note: Data presented as mean ± standard deviation (SD) for continuous variables or *n* (%) for categorical variables. BMI = Body Mass Index; BFP = Body Fat Percentage; ESE = Exercise Self-Efficacy; SE = Self-Esteem; MVPA = Moderate-to-Vigorous Physical Activity. *P*-value: Independent samples *t*-test was used for comparisons of continuous variables (age, height, weight, fat-free mass, body fat mass, BFP, BMI, ESE, SE, MVPA) between intervention and control groups. Chi-square test was used for categorical variables comparison (gender). A *P*-value < 0.05 was considered statistically significant.

**Table 2:** Hierarchical regression model predicting Post-intervention BFP (T1) for the IG.

Model	Variables	B	SE	β	t	*P*	95% CI	VIF
Model 1	(Constant)	6.93	5.32		1.30	0.200	[−3.80, 17.65]	
	Age	−0.35	0.35	−0.05	−1.02	0.316	[−1.06, 0.35]	1.08
	Gender	−0.85	0.61	−0.07	−1.38	0.174	[−2.08, 0.39]	1.42
	Baseline BFP (T0)	0.87	0.05	0.93	18.61	< 0.001	[0.78, 0.97]	1.36
Model 2	(Constant)	7.28	3.88		1.88	0.068	[−0.55, 15.11]	
	Age	−0.39	0.25	−0.05	−1.54	0.130	[−0.90, 0.12]	1.14
	Gender	−0.36	0.44	−0.03	−0.80	0.427	[−1.25, 0.54]	1.50
	Baseline BFP (T0)	0.93	0.04	0.99	25.31	< 0.001	[0.85, 1.00]	1.67
	$\:{\Delta\:}$ESE	0.01	0.01	0.02	0.57	0.572	[−0.02, 0.04]	1.42
	$\:{\Delta\:}$SE	−0.22	0.05	−0.16	−4.24	< 0.001	[−0.32, −0.12]	1.55
	$\:{\Delta\:}$MVPA	−0.13	0.06	−0.08	−2.06	0.046	[−0.26, −0.00]	1.62

Note: Dependent Variable: Post-intervention BFP (T1). Model 1 adjusted for demographics and baseline BFP (*R*^2^ = 0.918). Model 2 added change scores (Δ*R*^2^ = 0.044, *P* < 0.001). VIF = Variance Inflation Factor.

**Table 3:** Hierarchical regression model predicting Post-intervention BFP (T1) for the CG.

Model	Variables	B	SE	β	t	*P*	95% CI	VIF
Model 1	(Constant)	3.05	2.79		1.09	0.280	[−2.56, 8.66]	
	Age	0.05	0.19	0.01	0.27	0.791	[−0.33, 0.44]	1.03
	Gender	−0.08	0.32	−0.01	−0.26	0.795	[−0.73, 0.56]	1.33
	Baseline BFP (T0)	0.90	0.03	0.98	35.17	< 0.001	[0.85, 0.95]	1.35
Model 2	(Constant)	2.77	3.11		0.89	0.379	[−3.51, 9.04]	
	Age	0.06	0.20	0.01	0.30	0.764	[−0.34, 0.46]	1.09
	Gender	−0.16	0.37	−0.01	−0.43	0.669	[−0.91, 0.59]	1.80
	Baseline BFP (T0)	0.91	0.03	0.99	29.52	< 0.001	[0.85, 0.97]	1.94
	$\:{\Delta\:}$ESE	−0.00	0.01	−0.00	−0.07	0.948	[−0.02, 0.02]	1.53
	$\:{\Delta\:}$SE	−0.07	0.05	−0.04	−1.41	0.167	[−0.18, 0.03]	1.37
	$\:{\Delta\:}$MVPA	0.01	0.04	0.01	0.27	0.788	[−0.08, 0.10]	1.31

Note: Dependent Variable: Post-intervention BFP (T1). Model 1 adjusted for demographics and baseline BFP (*R*^2^ = 0.974). Model 2 added change scores (Δ*R*^2^ = 0.002, *P* = 0.467). VIF = Variance Inflation Factor.

## Supplementary Information

Below is the link to the electronic supplementary material.

Supplementary Material 1

## Data Availability

Datasets generated and analyzed during the current study are available from the corresponding author (Y.Z.) upon reasonable request.
