# Supplementary material for: The impact of exercise self-efficacy, self-esteem and physical activity on body fat percentage changes in adolescents during fat loss interventions
Source: Sci Rep. 2026 Jan 23;16:6049. doi: 10.1038/s41598-026-37238-y (PMC12902059; doi:10.1038/s41598-026-37238-y)
Supplement: Supplementary file 1 — Supplementary Material 1 [file 41598_2026_37238_MOESM1_ESM.docx]

**Intervention Protocol**

The weight loss intervention in this study comprised both exercise modalities and dietary restrictions, with the exercise component consisting of resistance training and aerobic conditioning.

1. **Resistance Training**

To ensure a 72-hour recovery period for the same muscle groups [1], the study implemented a three-way split resistance training regimen (anterior upper body, posterior upper body, and lower body). This program included a series of resistance exercises targeting each muscle group, with the subsequent brackets indicating regressions or progressions in the training modalities. Examples include:

1. Lower extremity extensors: Squats (standard/unilateral band resistance)

2. Core flexors: Crunches (band-assisted/standard/band-resisted)

3. Upper extremity compound movements: Push-ups (inclined/kneeling/standard/band-resisted)

4. Lower extremity flexors: Nordic hamstring curls (band-assisted/standard)

5. Core extensors: Supine back extensions (standard/band-resisted)

6. Upper extremity compound pull: Pull-ups (band-assisted low bar pull-ups/low bar pull-ups/band-assisted pull-ups/standard pull-ups)

7. Upper extremity extensors: Shoulder presses (band-resisted)

8. Lower body functional movement: 50-meter sprint intervals

Resistance exercise intensity was established at 70-80% of one-repetition maximum (1RM) [2]. This corresponded to bodyweight resistance exercises performed for 7-13 repetitions, with fewer than 7 repetitions indicating the necessity for regression, while achieving 13 or more repetitions with an intensity below 70% called for progression in the exercise. Each exercise was structured into 4 sets of 8-12 repetitions, with inter-set rest intervals of 60 seconds, in accordance with the principles of hypertrophy resistance training [3]. The duration of resistance training sessions was 30 minutes (from 4:30 PM to 5:00 PM). After every four-week cycle, a periodic assessment of maximal repetitions was conducted for load adjustments or progression, applying the following formula for calculating band-resisted training RM: 1RM = (resistance × repetitions / 30) + resistance [4].

1. **Aerobic Training**

Training intensity for aerobic conditioning was measured using the maximum heart rate paradigm, employing the formula 206.9 - (0.67 × age) to delineate an aerobic training intensity range of 57-67% of maximum heart rate [5]. Exercise intensity was closely monitored via a smart fitness tracker that recorded heart rate. Aerobic training sessions were designed to last 30 minutes (from 5:00 PM to 5:30 PM).

1. **Caloric Restriction Protocol**

For caloric restriction, a guided semi-structured dietary approach was employed due to the constraints of practical conditions and participant characteristics, which precluded closed-loop training and diet management. The Harris-Benedict equation [6] was utilized to estimate basal metabolic expenditure, which, when combined with activity levels, determined total caloric expenditure. A caloric ceiling of 75% of total expenditure was enforced, requiring participants' parents to adhere to this caloric upper limit and document daily caloric intake and food types. These records were regularly reviewed to ensure compliance with caloric guidelines. Additionally, a dietary assessment was conducted biweekly for all participants, which involved a four-day dietary recall encompassing three school days and one weekend day. To accurately estimate energy intake and facilitate recording, parents of participants were trained in utilizing caloric calculation software (Boohee) for calculating caloric intake and completing a 24-hour dietary recall questionnaire.

1. **References**

1. Regis R, Martim B, N W E, et al. Time course of strength and echo intensity recovery after resistance exercise in women. J Strength Cond Res. 2012;26(9).

2. J S B, Jozo G, Dan O, et al. Strength and hypertrophy adaptations between low- vs. high-load resistance training: a systematic review and meta-analysis. J Strength Cond Res. 2017;31(12).

3. A E I G, B V R, S S S R, et al. Effects of detraining on muscle strength and hypertrophy induced by resistance training: a systematic review. Muscles. 2022;1(1).

4. Guo H. Study on prediction equations to estimate one repetition maximum of young adults [dissertation]. Tianjin University of Sport; 2012.

5. Christopher K, Kris B, John N, et al. Ratings of perceived exertion of ACSM exercise guidelines in individuals varying in aerobic fitness. Res Q Exerc Sport. 2006;77(1).

6. Curtis B. Chart for the computation of Harris-Benedict standards of basal metabolism. N Engl J Med. 1930;202(11).
